# The potential DNA methylation markers of cardiovascular disease in patients with type 2 diabetes

**DOI:** 10.1186/s12920-023-01689-3

**Published:** 2023-10-12

**Authors:** Yunbiao He, Xia Chen, Mingliang Liu, Lei Zuo, Zhiyu Zhai, Long Zhou, Guangzhen Li, Li Chen, Guolong Qi, Chunxia Jing, Guang Hao

**Affiliations:** 1 Department of Public Health and Preventive Medicine, School of Medicine, Jinan University, 601 West Huangpu Road, Guangzhou, 510632 Guangdong China; 2 Department of Medicine, Medical College of Georgia, Georgia Prevention Institute, Augusta University, Augusta, GA USA; 3 Guangdong Key Laboratory of Environmental Exposure and Health, Jinan University, Guangzhou, China; 4 Department of Epidemiology, School of Medicine, Jinan University, 601 West Huangpu Road, Guangzhou, 510632 Guangdong China

**Keywords:** Type 2 diabetes, Cardiovascular disease, Methylation, Biomarker, The Han Chinese

## Abstract

**Background:**

DNA methylation is associated with cardiovascular (CV) disease. However, in type 2 diabetes (T2D) patients, the role of gene methylation in the development of CV disease is under-studied. We aimed to identify the CV disease-related DNA methylation loci in patients with T2D and to explore the potential pathways underlying the development of CV disease using a two-stage design.

**Methods:**

The participants were from the Jinan Diabetes Cohort Study (JNDCS), an ongoing longitudinal study designed to evaluate the development of CV risk in patients with T2D. In the discovery cohort, 10 diabetic patients with CV events at baseline were randomly selected as the case group, and another 10 diabetic patients without CV events were matched for sex, age, smoking status, and body mass index as the control group. In 1438 T2D patients without CV disease at baseline, 210 patients with CV events were identified after a mean 6.5-year follow-up. Of whom, 100 patients who experienced CV events during the follow-up were randomly selected as cases, and 100 patients who did not have CV events were randomly selected as the control group in the validation cohort. Reduced representation bisulfite sequencing and Targeted Bisulfite Sequencing were used to measure the methylation profiles in the discovery and validation cohort, respectively.

**Results:**

In the discover cohort, 127 DMRs related to CV disease were identified in T2D patients. Further, we validated 23 DMRs mapped to 25 genes, of them, 4 genes (*ARSG, PNPLA6, NEFL*, and *CRYGEP*) for the first time were reported. There was evidence that the addition of DNA methylation data improved the prediction performance of CV disease in T2D patients. Pathway analysis identified some significant signaling pathways involved in CV comorbidities, T2D, and inflammation.

**Conclusions:**

In this study, we identified 23 DMRs mapped to 25 genes associated with CV disease in T2D patients, of them, 4 DMRs for the first time were reported. DNA methylation testing may help identify a high CV-risk population in T2D patients.

**Supplementary Information:**

The online version contains supplementary material available at 10.1186/s12920-023-01689-3.

## Introduction

Cardiovascular (CV) disease, accounting for 52% of deaths, is the main cause of death in patients with type 2 diabetes (T2D). [1] People with T2D are two to six times more likely to die from CV disease than those without T2D. [2, 3] Long-term hyperglycemia is strongly associated with macrovascular complications and microvascular complications (such as kidney diseases, retinopathy, and nervous system diseases). [4] In severe cases, hyperglycemia can even lead to blindness, renal failure, deterioration of life quality, and even death. [5] Even after glycemic control is achieved, patients with T2D continue to increase inflammation and vascular problems. [6]

Burgeoning evidence suggests that epigenetic modifications may significantly derail transcriptional programs implicated in angiogenesis, oxidative stress and inflammation. [7] DNA methylation is a major epigenetic modification involving the addition of a methyl group to the 5 position of cytosine by DNA methyltransferase to form 5-methylcytosines. [8] There is increasing evidence that DNA methylation plays a vital role in the development of CV events. [9, 10]. For example, in a European prospective cohort study, it was reported that the methylation level of the *ABCG1* gene was positively correlated with the risk of CV disease. [11] Carraro et al. found that there was a significant correlation between the high methylation level of the *SERPINE1* gene and several cardiac metabolic indexes (waist circumference, waist-hip ratio, and uric acid). [12] Aberrant DNA methylation represents one of key determinants of vascular lesions and, thus, putative useful biomarkers for prevention and diagnosis of CV risk in diabetics. [13] In a recent pilot study, Benincasa et al. reported that *SPARC* hypomethylation in CD08 + T cells may be a useful biomarker of vascular complications in pre-diabetics patients. [14] However, the role of gene methylation in the development of CV disease need to be further investigated.

Growing evidence has demonstrated that network medicine is a promising molecular-bioinformatic approach to identify the signaling pathways underlying the pathogenesis of CV disease in patients with T2D. [13, 15, 16] The purpose of this study was to identify the CV disease-related DNA methylation loci in patients with T2D and to explore the potential pathways underlying the development of CV disease using network approaches.

## Methods

### Study subjects

The participants were from the Jinan Diabetes Cohort Study (JNDCS), an ongoing longitudinal study designed to evaluate the development of CV risk in patients with T2D. A total of 2756 patients were continuously recruited between 2012 and 2017 from the First Affiliated Hospital of Jinan University, Guangzhou, China. All patients were diagnosed according to the 2003 American Diabetes Association [17].

Demographic information was collected by standardized questionnaire, and physical measurements and laboratory test results were extracted from the hospital medical record system. The venous blood (5 mL) was drawn in the morning and stored at -70 ºC. All patients were followed up by telephone calls in 2021, to collect information on CV events, including coronary artery disease, myocardial infarction, percutaneous coronary angioplasty and/or stenting, coronary artery bypass grafting, heart failure, and CV death.

The study was approved by the Institutional Review Board of Jinan University, and all participants provided written informed consent.

### Study design

The prevalence of CV disease was 50% (1378/2756) at baseline. In the discovery cohort, 10 diabetic patients with CV events were randomly selected as the case group, and another 10 diabetic patients without CV events were matched for sex, age (± 1 years), smoking status, and body mass index (± 0.5 kg/m^2^) as the control group. Reduced representation bisulfite sequencing (RRBS) was used to measure the methylation profiles. The selection principle of methylated fragments for validation were as follows: (1) The results of GO enrichment and KEGG enrichment [18]; (2) Literature review; (3) Significantly different methylation sites. The details were provided **in Supplementary File 1.** (**Figure ****S1****-****S2**** and Table ****S1****-S3**)

In 1438 T2D patients without CV disease at baseline, 210 patients with CV events were identified after a mean 6.5-year follow-up. Of whom, 100 patients who experienced CV events during the follow-up were randomly selected as cases, and 100 patients who did not have CV events were randomly selected as the control group (a nested case-control design) in the validation cohort. (**Figure S3 in Supplementary File 1**)

### Reduced representation bisulfite sequencing (RRBS)

Genomic DNA was extracted from peripheral whole blood using DNeasy Blood & Tissue Kit (Qiagen). RRBS library was prepared using the Acegen Rapid RRBS Library Prep Kit (Acegen, Cat. No. AG0422). In brief, 100 ng of genomic DNA was digested with MspI, end-repaired, 3’-dA-tailed, and ligated to 5-methylcytosine-modified adapters. After bisulfite treatment, the DNA was amplified with 12 cycles of PCR using Illumina 8-bp dual index primers. Size selection was performed to obtain DNA fractions of MspI-digested products in the range of 100–350 bp using a dual-SPRI® protocol according to the manufacturer’s protocol. The constructed RRBS libraries were then analyzed by Agilent 2100 Bioanalyzer and finally sequenced on Illumina platforms using a 150 × 2 paired-end sequencing protocol. [19–21].

### Targeted Bisulfite sequencing (TBS)

We used trimming to truncate the sequencing adapters and low-quality data of the sequencing data and obtain clean data for subsequent analysis. Trimmomatic (version 0.36) software was used for raw data trimming. Using the sliding window method, 4 bases are a window. If the average base quality value of the window is lower than 15, the reads will be truncated there. Next, the clean data was aligned with the amplified target sequence, and the BSMAP 2.73 software was used for alignment. The alignment mode was mapped to 2 forward strands, i.e. BSW (++) and BSC (-+). After the alignment was completed, the methylation level of the CG site was calculated using the python program for calculating methylation that comes with BSMAP. The calculation principle is Methyl value = C-reads / (C-reads + T-reads) * 100%, where C-reads is the number of methylation-supporting reads covering the site (the site is measured as C reads), T-reads is the number of reads that do not support methylation covering the site (reads with a T at the site). [22, 23].

### Statistical analysis

Continuous variables were expressed as means ± standard deviation, and the mean values of the two groups are compared by student’s *t*-test. Categorical variables were reported as percentages (n [%]), and the *χ*^2^ test was used to test differences between groups. The DNA methylation rates in DMRs and CpG sites between the two groups were compared by student’s *t*-test, and an FDR < 0.05 was considered validated successfully. Moreover, random forests were used to evaluate the variable importance of CV risk factors. The area under the receiver operating characteristic curve (AUC) was estimated to assess the potential predictive value of DNA methylation data.

R (version 4.0.5) package “org.Hs.eg.db” (version 3.12.0) and “clusterProfiler” (version 4.4.4) were used for GO-BP biological process analysis, GO-MF molecular function analysis, GO-CC cytological component analysis, and KEGG signaling pathway analysis [18]. In the process of GO enrichment analysis, functional items were selected from the results of GO enrichment and plotted according to the value of *P* < 0.05. In the process of KEGG enrichment analysis, the parameter was set as *P* < 0.05. We also performed Protein-protein interaction (PPI) Network analysis for DMR along the sequencing direction of RRBS and TBS, respectively. String [24] (https://string-db.org/) is one of the databases of protein-protein interaction networks that enables the analysis of known proteins and the prediction of proteins with possible biological effects. The connection between the protein network was set as reliability, and the minimum action score was set as 0.400. The isolated or scattered nodes were removed before retrieval analysis and graph drawing. Further, we utilized a network visualization tool ‘Cytoscape’ [25] for the visualization of the network where nodes denote proteins and edges denote the connections between the nodes, and the genes we uploaded were filled in yellow.

All analyses were performed using Stata software version 12 (STATA Corp., TX, US) and R 4.0.5 (R Foundation for Statistical Computing Vienna, Austria).

## Result

### Characteristics of study participants

The basic characteristics and laboratory examination indices for the discovery and validation cohort were presented in Table 1. There are no statistical differences between the case and control groups in the discovery cohort. For the validation cohort, there was a statistical difference in educational attainment, urine protein, and carotid atherosclerosis (*P* < 0.05).

**Table 1:** Baseline characteristics of the study population

Characteristics	RRBS				TBS		
	Controls (n = 10)	Cases (n = 10)	*P*-value		Controls (n = 100)	Cases (n = 100)	*P*-value
Gender, n (%)			1.000				1.000
Male	3 (30.00)	3 (30.00)			39 (39.00)	39 (39.00)	
Female	7 (70.00)	7 (70.00)			61 (61.00)	61 (61.00)	
Age (years)	59.50 ± 10.63	59.50 ± 10.63	1.000		64.79 ± 10.74	64.78 ± 10.77	0.995
Married, n (%)	10 (100.00)	10 (100.00)			95 (95.00)	97 (97.00)	0.470
Education attainment, n (%)			0.645				**0.005**
Less than primary school	4 (40.00)	3 (30.00)			55 (55.00)	43 (43.00)	
Middle school	4 (40.00)	6 (60.00)			20 (20.00)	41 (41.00)	
Tertiary school or higher	2 (20.00)	1 (10.00)			25 (25.00)	16 (16.00)	
Occupation, n (%)			0.121				0.201
Retiree	1 (10.00)	4 (40.00)			50 (50.00)	59 (59.00)	
Others	9 (90.00)	6 (60.00)			50 (50.00)	41 (41.00)	
Body mass index (kg/m2)	25.33 ± 3.38	25.76 ± 3.91	0.793		24.09 ± 3.03	25.03 ± 3.86	0.057
Smokers, n (%)	0 (0.00)	0 (0.00)			16 (16.00)	26 (26.00)	0.083
Drinkers, n (%)	0 (0.00)	0 (0.00)			5 (5.00)	8 (8.00)	0.390
Laboratory results							
Fast blood-glucose (mmol/L)	9.03 ± 3.15	8.57 ± 3.04	0.743		9.86 ± 4.66	8.69 ± 4.69	0.101
Postprandial blood glucose (mmol/L)	17.16 ± 4.73	15.13 ± 3.95	0.310		15.57 ± 5.86	14.51 ± 5.77	0.228
HbA1c (%)	8.77 ± 2.92	8.65 ± 2.25	0.929		9.12 ± 2.74	8.71 ± 2.67	0.309
Fasting C-peptide (ng/ml)	1.52 ± 0.84	1.74 ± 1.14	0.733		1.67 ± 1.50	1.80 ± 1.51	0.605
1 h postprandial, C-peptide (ng/ml)	4.57 ± 4.00	4.75 ± 4.91	0.960		3.38 ± 2.82	3.44 ± 2.82	0.921
2 h postprandial, C-peptide (ng/ml)	3.16 ± 1.82	6.21 ± 7.34	0.308		4.15 ± 3.40	4.71 ± 3.77	0.330
Urinary creatinine (µmol/L)	10712.75 ± 6879.51	6726.25 ± 4451.13	0.190		7408.81 ± 4108.92	7770.75 ± 4797.89	0.619
Urinary microalbumin (µg/mL)	152.25 ± 382.83	136.05 ± 331.71	0.929		198.42 ± 697.97	64.01 ± 152.52	0.095
Urinary protein (g/L)					2.92 ± 3.49	0.21 ± 0.12	**0.025**
GFR (ml/min)	86.70 ± 24.97	62.86 ± 19.38	0.091		73.81 ± 18.67	75.00 ± 16.43	0.721
TC (mmol/L)	4.95 ± 1.40	5.02 ± 0.99	0.906		5.03 ± 1.35	4.93 ± 1.11	0.576
TG (mmol/L)	2.54 ± 1.72	1.41 ± 0.53	0.062		1.92 ± 1.81	1.94 ± 1.27	0.939
HDL (mmol/L)	1.11 ± 0.20	1.10 ± 0.21	0.914		1.12 ± 0.27	1.10 ± 0.29	0.587
LDL (mmol/L)	2.48 ± 0.87	3.12 ± 0.73	0.091		2.97 ± 1.02	2.93 ± 0.97	0.784
ALT (U/L)	25.70 ± 8.57	21.70 ± 10.57	0.365		29.73 ± 28.43	37.33 ± 102.37	0.490
Blood creatinine (µmol/L)	63.30 ± 11.75	61.30 ± 11.03	0.699		75.11 ± 47.57	73.48 ± 39.31	0.794
Blood uric acid (µmol/L)	364.3 ± 135.95	337.7 ± 72.23	0.592		372.09 ± 119.21	373.12 ± 122.23	0.953
hsCRP (mg/L)	4.45 ± 1.76	5.93 ± 5.19	0.734		7.95 ± 14.37	24.94 ± 59.14	0.221
Comorbidities							
Hypertension, n (%)	5 (50.00)	6 (66.67)	0.463		60 (60.00)	67 (67.00)	0.304
Hyperlipemia, n (%)	2 (20.00)	5 (50.00)	0.160		36 (36.00)	39 (39.00)	0.661
Carotid atherosclerosis, n (%)	9 (90.00)	10 (100.00)			66 (70.21)	84 (91.30)	**< 0.001**
Reduced left ventricular diastolic function, n (%)	6 (66.67)	7 (87.50)	0.312		62 (70.45)	67 (81.71)	0.087
Peripheral neuropathy, n (%)	2 (25.00)	2 (28.57)	0.876		10 (14.93)	14 (28.00)	0.083

Continuous variables are presented as mean ± standard deviation, and categorical variables are presented as cases (percentage)

Abbreviation: HbA1c: glycated hemoglobin |glycosylated hemoglobin; GFR: Glomerular filtration rate; TC: total cholesterol; TG: Triglycerides; HDL: High-density lipoprotein; LDL: Low-density lipoprotein; ALT: Alanine aminotransferase; hs-CRP: hypersensitive C-reactive protein

### RRBS in the discovery cohort

**Fig. 1:**
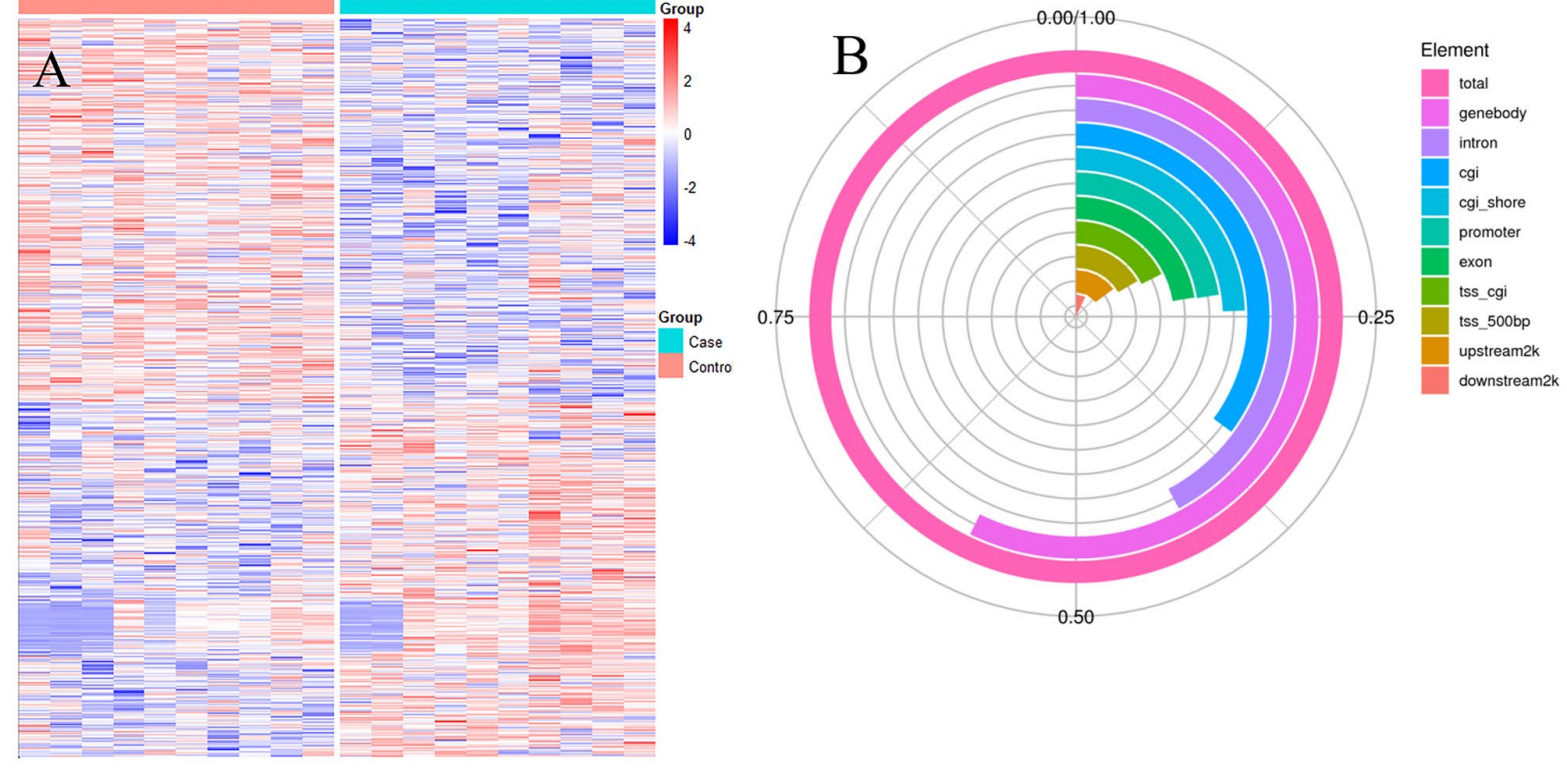
The distribution of differentially methylated sites (DMRs) using reduced representation bisulfite sequencing (RRBS) in the discovery cohort **A**: The heatmap of DMRs **B**: The location of DMRS position

Using the next generation RRBS, a total of 20,259 DMRs were measured, of them, 12,981 DMRs were significantly different between T2D patients with and without CV disease (FDR < 0.01) (6382 decreased, 6599 increased; **Table S4 in Supplementary File 2**). (Fig. 1A). There were 57.19% of the methylation sites located in the gene body and 22.57% located in the promoter (Fig. 1B **and Table S6 in Supplementary File 2**). According to the results from GO enrichment, KEGG enrichment, and literature review, we initially selected 127 DMRs for further validation (**Supplementary File 2 for more details**). (**Table S7 in Supplementary File 2**).

### TBS in the validation cohort

**Fig. 2:**
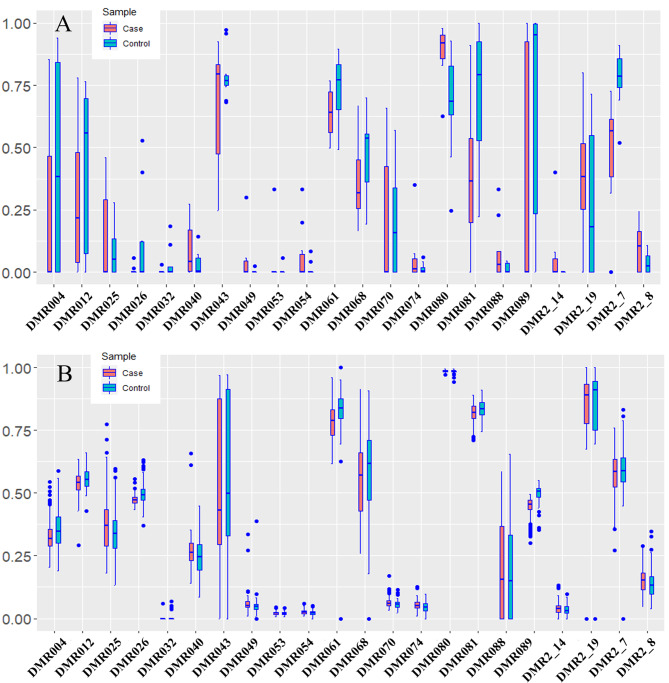
A total of 23 validated differentially methylated sites (DMRs) **A**: 23 DMR in the discovery cohort **B**: 23 DMR in the validation cohort

Of 127 DMRs, the bisulfite sequencing primers in 87 DMRs were successfully designed for TBS analysis. A total of 23 DMRs with 25 genes were further validated (Fig. 2**and Table S7-9 in Supplementary File 2**). Twelve genes were hypermethylated (*LMF1*, *FZD5*, *COL6A1*, *TBX1*, *CACNA1D, PTPRN2, NEFL, RXRA, G6PD, IKBKG, ADCY6* and *WNT7A* genes), and 13 genes were hypomethylation (*PIK3CD, PDE4DIPP1, H19, MIR675, ARSG, PNPLA6, CRYGEP, TNIP1, PON1, COL5A1, KDM6A, CREB5* and *SERPINE1*). Of them, 4 genes (*ARSG*, *PNPLA6*, *NEFL*, and *CRYGEP*) were reported for the first time. (**Table S9 in Supplementary File 2**)

### Predictive value of the identified methylation sites

**Fig. 3:**
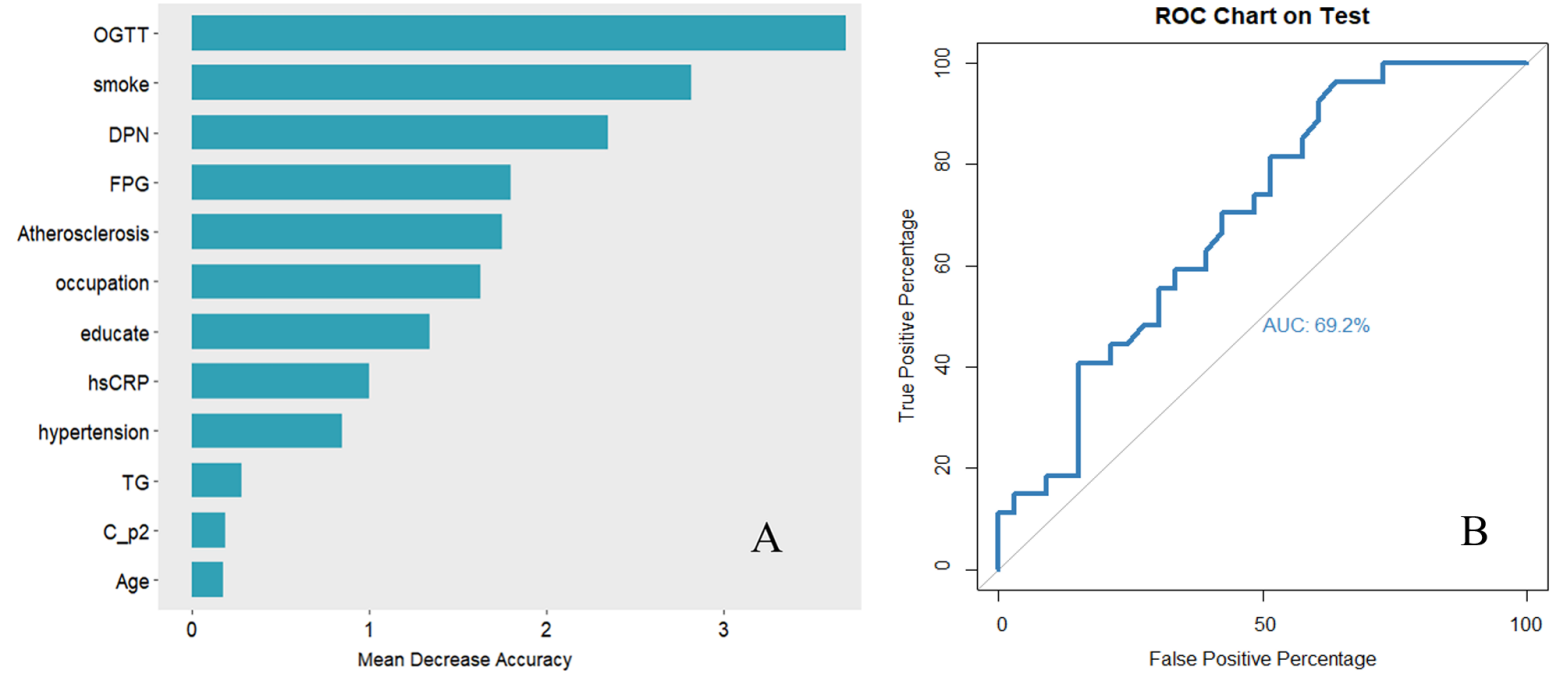
The baseline predictive model using the top 9 ranked important variables **A**: The importance ranking of selected variables using random forest **B**: ROC curves of the predictive model with top 9 ranked important variables (postprandial blood glucose, smoking status, peripheral neuropathy, fast blood-glucose, carotid atherosclerosis, occupation, education attainment, high sensitivity c-reactive protein, and hypertension)

**Fig. 4:**
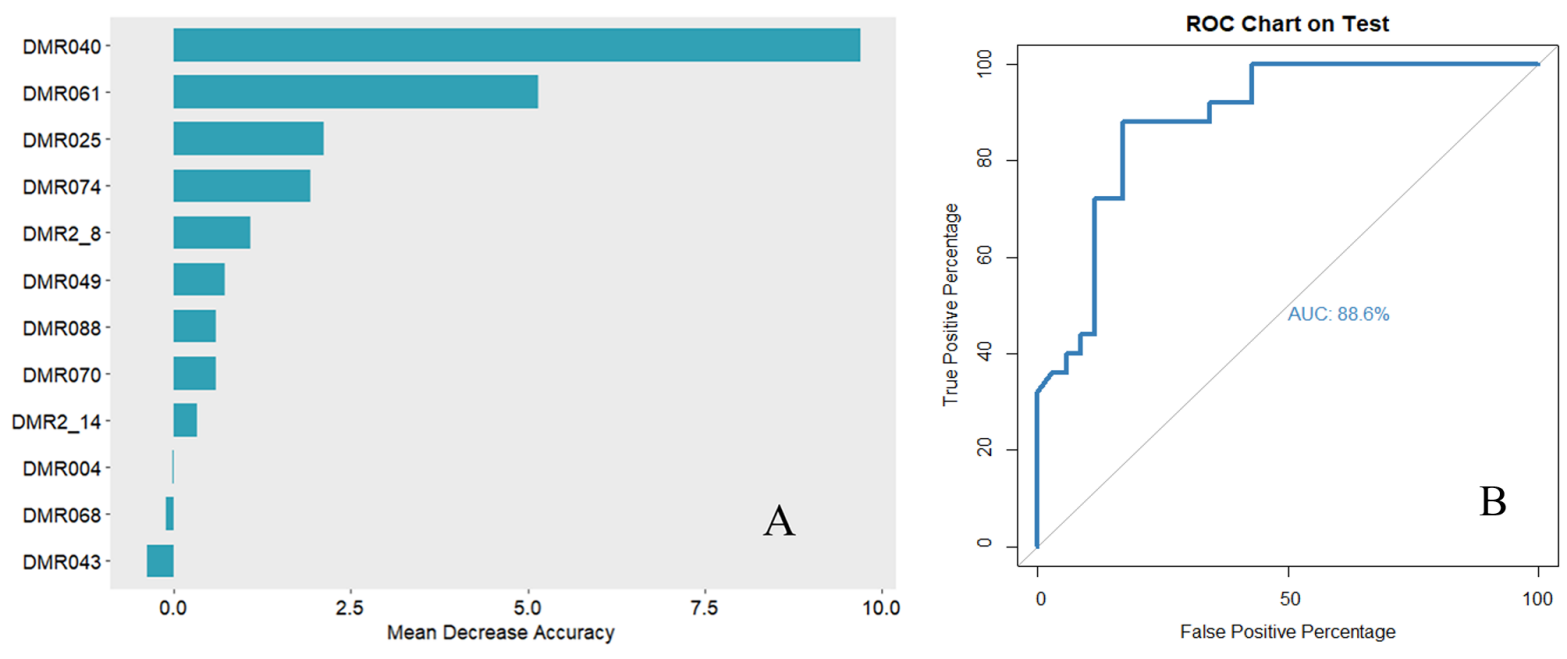
The predictive model plus using the top 9 ranked important variables and top 4 ranked important DMRs **A**: The importance ranking of significant DMRs using random forest **B**: ROC curves of the predictive model using the top 9 ranked important variables (postprandial blood glucose, smoking status, peripheral neuropathy, fast blood-glucose, carotid atherosclerosis, occupation, education attainment, high sensitivity c-reactive protein, and hypertension) plus top 4 ranked important DMRs (DMR025, DMR061, DMR040, and DMR074)

**Fig. 5:**
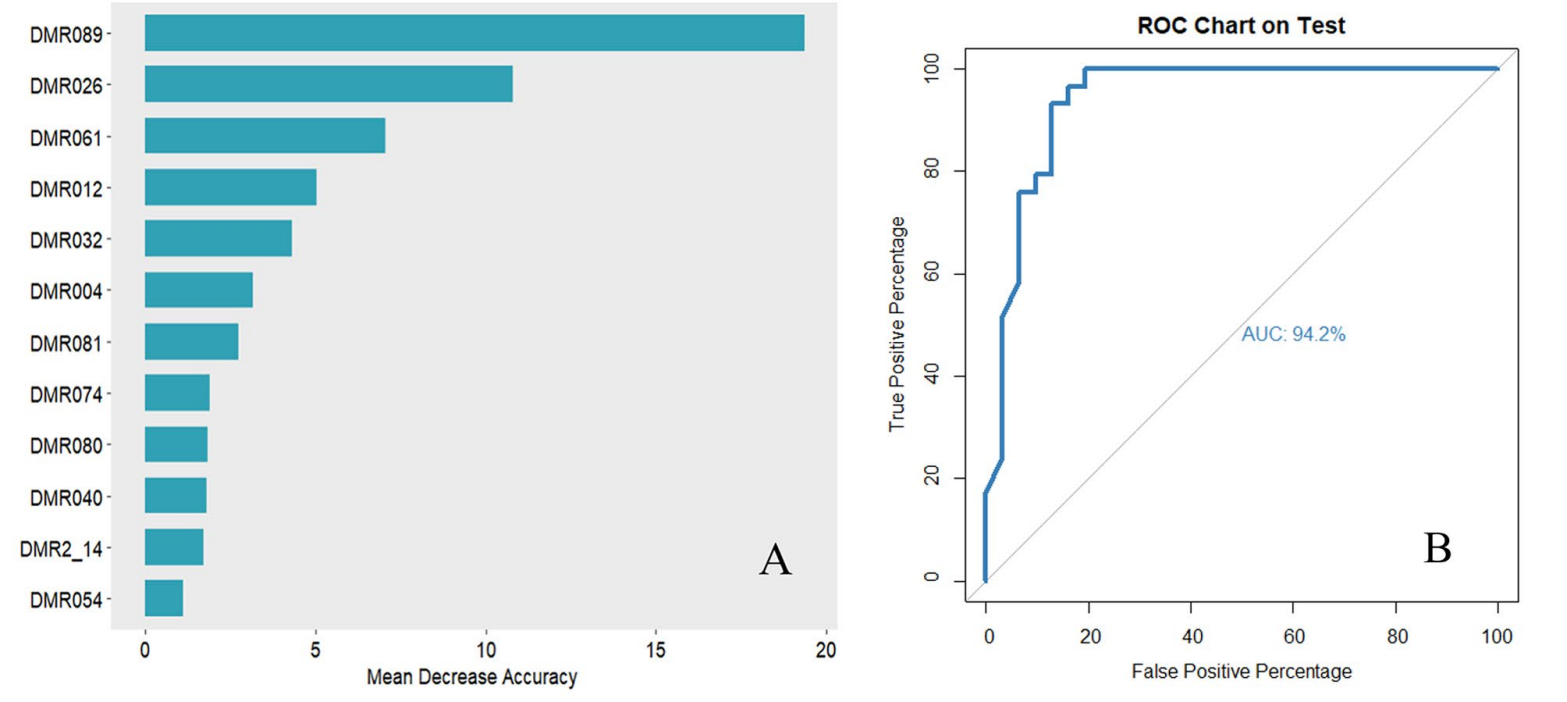
The predictive model plus using the top 9 ranked important variables and top 4 ranked important CpGs **A**: The importance ranking of significant DMRs using random forest **B**: ROC curves of the predictive model using the top 9 ranked important variables (postprandial blood glucose, smoking status, peripheral neuropathy, fast blood-glucose, carotid atherosclerosis, occupation, education attainment, high sensitivity c-reactive protein, and hypertension) plus top 4 ranked important CpGs located in DMR012, DMR026, DMR061, and DMR089

A total of 32 potential variables in Table 1 were analyzed by random forest, of them, 9 variables (postprandial blood glucose, smoking status, peripheral neuropathy, fast blood-glucose, carotid atherosclerosis, occupation, education attainment, high sensitivity c-reactive protein (hsCRP), and hypertension) with Mean Decrease Accuracy value > 0.5 were included in the prediction model, and the AUC was 69.2% (Fig. 3). When further adding the DMRs with the top 4 importance ranking (including DMR for *LMF1* and *SOX8*, DMR for *TNIP1*, and DMR for *FZD5*) to the model, the prediction performance of the model was improved substantially, and its AUC reached 88.6% (Fig. 4). Further, when the top four methylation sites (including methylation sites of *MIR675*, *ARSG*, *TNIP1*, and *KDM6A*) were added to the model, the prediction performance of the model was significantly improved (AUC = 94.2%). **(**Fig. 5**)**

### Pathway analysis and GO enrichment analysis

**Fig. 6:**
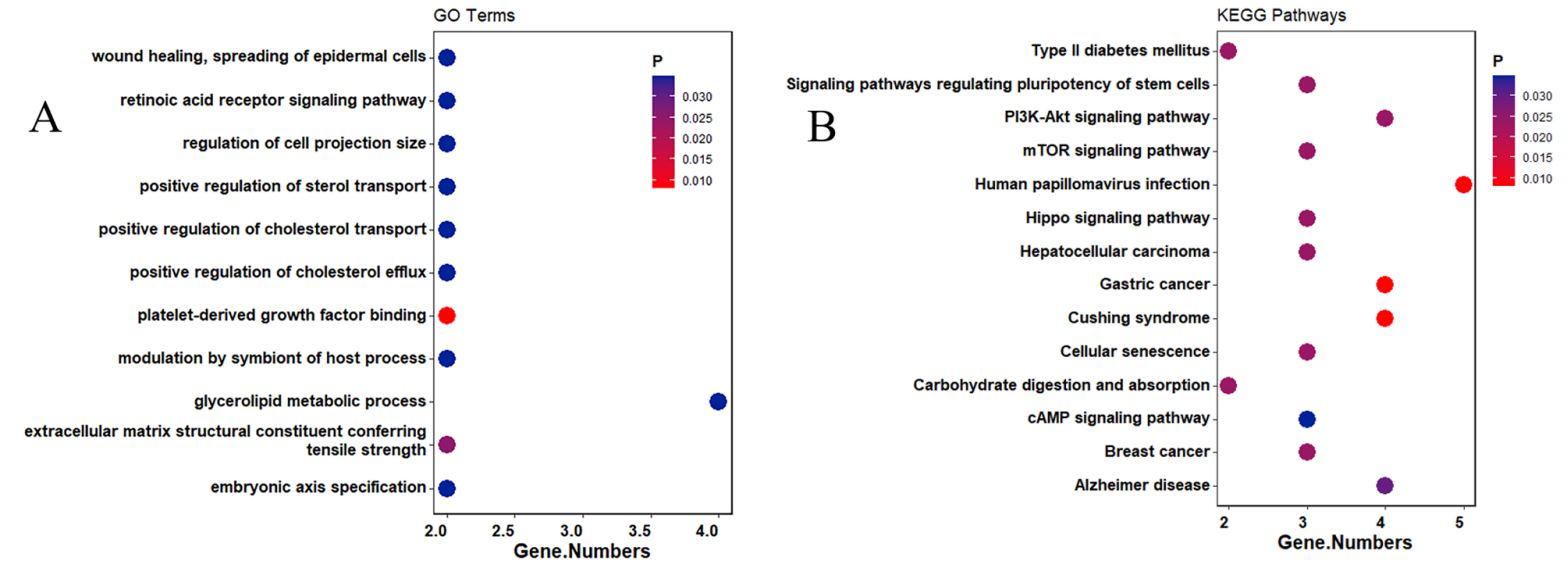
Pathway enrichment map of validated methylation sites **A**: Scatter plot of GO enrichment analysis **B**: Scatter plot of KEGG enrichment analysis

**Fig. 7:**
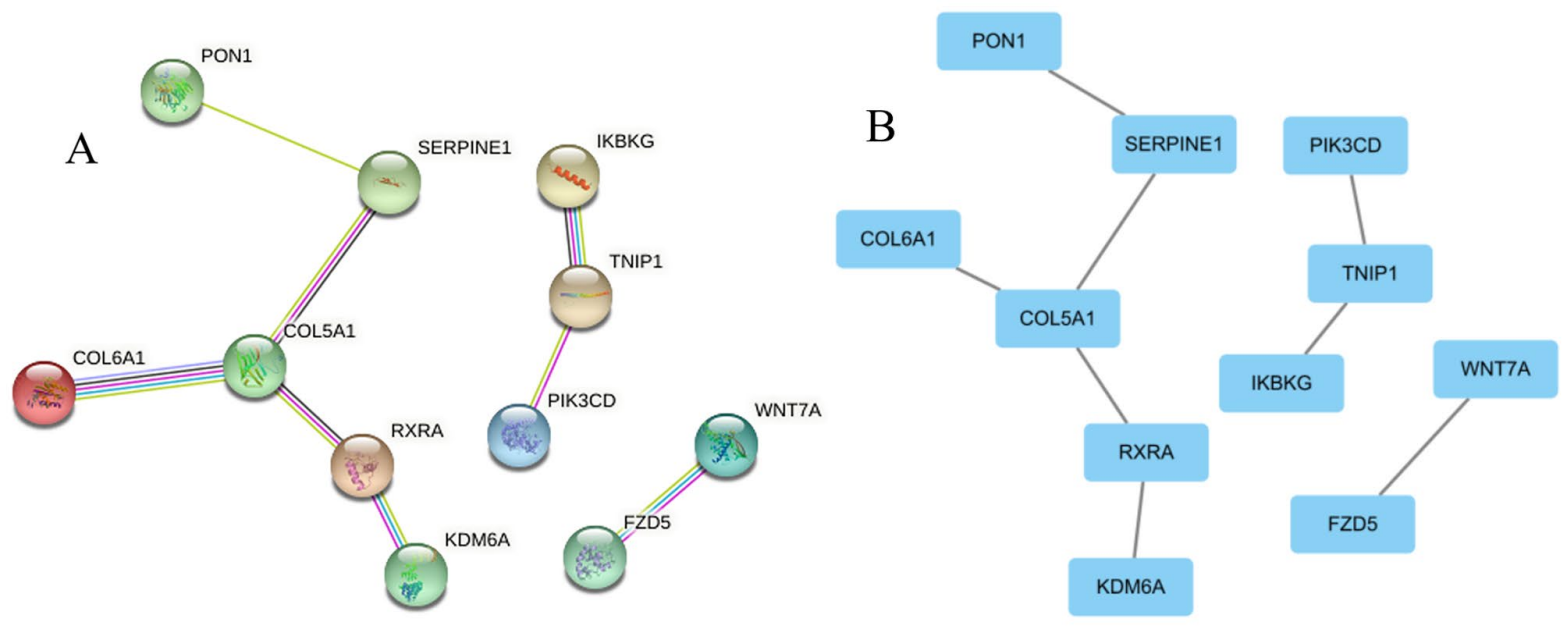
Schematic representation of spatial and planar interactions of proteins expressed by genes **A**: Spatial structure diagram of the gene-expressed proteins **B**: Planar structure diagram of the gene-expression

The genes corresponding to the validated DMRs were analyzed by GO and KEGG enrichment, and yielded significant (FDR < 0.05) enrichment of 22 KEGG and 17 GO pathways (Fig. 6, **Table S10 and S11 in Supplementary File 2**). These pathways are involved in CV comorbidities (such as Type II diabetes mellitus, Alzheimer’s disease, Cushing syndrome, and Dilated cardiomyopathy), cancers (such as gastric cancer, breast cancer, hepatocellular carcinoma), and the inflammatory pathway (such as signaling pathways regulating pluripotency of stem cells, mTOR signaling pathway, hippo signaling pathway, pi3k-Akt signaling pathway, and cAMP signaling pathway). Further, there were 25 genes located in the 23 DMRs used for PPI enrichment analysis. (**Table S9 in Supplementary File 2**). The proteins in this network were related to some signaling pathways, such as mTOR signaling pathway, cellular senescence, hippo signaling pathway, and type 2 diabetes mellitus. **(**Fig. 7**)**

## Discussion

DNA methylation plays a critical role in the development of CV disease. It is well-known that patients with T2D are at higher risk for CV disease than those without, but the role of DNA methylation in T2D patients with CV is under-studied. In this study, we identified 23 DMRs mapped to 25 genes associated with CV disease in T2D patients, of them, 4 genes (*ARSG, PNPLA6, NEFL*, and *CRYGEP*) for the first time were reported. DNA methylation testing may help identify a high CV-risk population in T2D patients.

Most identified genes in our study were associated with CV disease and T2D. Studies have shown that the *LMF1* gene is involved in the regulation of lipase activity and metformin increased *LMF1* expression in the heart, suggesting that stimulation of *LMF1* may play a part in its TG-lowering action. [26, 27] Consistently, we found that the methylation level of the *LMF1* gene promoter significantly increased in T2D patients with CV disease. It is reported that SOX8 proteins were markedly increased in patients with heart failure. [28] In line with our results, *SOX8* gene body methylation was hypermethylated. The methylation of the *FZD5* gene promoter was increased in T2D patients with CV disease, which corroborates the findings of another study showing the involvement of *FZD5* in regulating diabetic vasculopathy. [29] Gene polymorphism of *TNIP1* was associated with coronary heart disease in the Chinese Han population, [30] and we observed a decrease in *TNIP1* gene body methylation in T2D patients with CV disease. The methylation levels of *NEFL* gene promoter in T2D patient with CV disease was elevated. Similarly, the study by Yadi et al. showed that the *NEFL* gene is involved in the process of the protective effect of cardiac insufficiency. [31] However, the relationship between NEFL and CV disease is still unclear and further studies are needed.

We, for the first time, identified 4 novel DMRs with 4 genes (*ARSG, PNPLA6, NEFL*, and *CRYGEP*) in T2D patients. Inflammation plays a critical role in the genesis, progression, and the manifestation of CV disease. [32, 33] NEFL (neurofilament light chain) is a neuronal cytoplasmic protein highly expressed in large calibre myelinated axons. [34] NEFL is considered as a potential biomarker for diverse neurological diseases, such as Alzheimer’s disease and frontotemporal dementia. [35, 36] NEFL was reported to activate the mTOR signaling pathway. [34, 37] Many studies showed that mammalian target of rapamycin (mTOR) signaling plays an important role in the general and inflammation-driven mechanisms that are related to the CV disease. [38, 39] The *CRYGEP* gene is considered to be a pseudogene with no evidence of expression in humans. Nevertheless, the gene remains largely intact and is at least potentially involved in gene conversion and even reactivation of the active gene. [40] It was suggested that the *CRYGEP* gene methylation level may affect the magnitude of Bacillus Calmette–Guérin immune responses. [41] Patatin-like phospholipase domain-containing protein 6 (PNPLA6) belongs to a family of hydrolases with at least eight members in mammals that react with different substrates such as phospholipids, triacylglycerols, and retinol esters. [42] PNPLA6 preferably hydrolyzes phosphatidylcholine (PC) and lysophosphatidylcholine (LPC). [43] LPC could be a messenger by signaling through membrane receptors. It was expected that PNPLA6 contains domains that are predicted to bind cAMP, [44] and the cAMP signaling pathway plays a key role in the regulation of cardiac function. [45] Consistently, our pathway analyses also yielded significant enrichment in the mTOR signaling pathway and cAMP signaling pathway. These genes have not been well-studied but the results provide some clues for future research directions.

This is among the first study to investigate the role of DNA methylation in T2D patients with CV disease. One strength is that a nested case-control design was used in the validation cohort. Another strength is that taking advantage of RRBS, we specifically analyzed DMRs rather than the methylation level of single CpG dinucleotides. DMRs can control spatiotemporal gene expression, have the most statistical power and by-pass putative effects of genetic polymorphisms during epigenome-wide association studies. [14] However, this study has some limitations. First, different tests are used in the discovery and validation phases, and not all bisulfite sequencing primers of identified DMRs were successfully designed for validation, which may miss new sites. Second, the lifestyles of T2D patients may have changed after diagnosis. Third, the patients in this study come from the Han nationality in southern China, so extrapolating the results to other populations should be cautious. Fourth, this study is limited by a small sample size, so future studies will benefit from the confirmation of these results in larger sample sizes. Finally, blood-based methylation signatures should be validated in cardiac tissues to advance our knowledge about the progression of CV diseases in patients with T2D.

## Conclusion

In this study, we validated 19 DMRs mapped to 21 genes associated with CV disease in T2D patients, moreover, we identified 4 novel DMRs with 4 genes (*ARSG, PNPLA6, NEFL*, and *CRYGEP*). Consistently, Pathway analyses also found that the related pathways are involved in CV comorbidities, T2D, and inflammation. The differentially methylated genes identified in this study may be valuable biomarkers for the early detection of CV disease and may help improve treatment strategies, drug targets, and diagnostic activities to reduce the threat to human health from CV disease in T2D. More independent cohort studies are required to confirm the prediction value of the DNA methylation data for the high CV-risk population in T2D patients.

### Electronic supplementary material

Below is the link to the electronic supplementary material.

Supplementary Material 1

Supplementary Material 2

## Data Availability

The datasets analyzed during the current study are available at NCBI Sequence Read Archive (SRA) with Accession Number: PRJNA930169 (https://www.ncbi.nlm.nih.gov/bioproject). The other data generated in this study are fully reflected in the manuscript.

## Abbreviations

CV: cardiovascular
T2D: type 2 diabetes
RRBS: reduced representation bisulfite sequencing
TBS: Targeted Bisulfite Sequencing
