# Supplementary material for: The potential DNA methylation markers of cardiovascular disease in patients with type 2 diabetes
Source: BMC Med Genomics. 2023 Oct 12;16:242. doi: 10.1186/s12920-023-01689-3 (PMC10568935; doi:10.1186/s12920-023-01689-3)
Supplement: Supplementary file 1 — Supplementary Material 1 [file 12920_2023_1689_MOESM1_ESM.docx]

**Supplementary File 1**

**The result selection principle of RRBS**

**1. Methylation sites were selected for significant difference criteria.**

Significant differences in methylation sites were determined by adjusted *P*< 0.05, length of DMR >50, number of CPG islands >10, the absolute value of DIFF >0.2, and finally 30 DMR were eligible.

**2. Results of GO enrichment and KEGG enrichment.**

GO enrichment and KEGG enrichment analysis were performed, and 17 GO items and 19 KEGG pathways related to cardiovascular disease were identified (**Figure S1 and Figure S2, Table S1 and Table S2**). Accordingly, a total of 494 DMRs mapped to 196 genes were identified. Further, a total of 58 DMRs with adjusted *P* value <0.05 and absolute value of foldchange >2, or adjusted *P* value <0.05 and the absolute value of DIFF >0.2 were selected.

**3. Methylation sites that have been shown in the literature.**

We systematically searched PubMed and Embase to retrieve all the available literature on DNA methylation and cardiovascular disease published till February 2022. The keywords are as follows: (“DNA methylation” OR “methylation” OR “epigenomics” OR “hypermethylation” OR “hypomethylation”) AND (“cardiovascular disease” OR “coronary heart disease” OR “stroke” OR “myocardial infarction” OR “heart failure”). A total of 4527 studies were initially identified. We reviewed 12 relevant articles. Finally, we further identified 24 potential DMRs (adjusted *P* <0.05) (**Table S3**).

Table S1. GO enrichment pathways

| Term | Term Name | Foldchange | *P* |
| --- | --- | --- | --- |
| GO:0001525 | angiogenesis | 2.17 | 0.0000 |
| GO:0007596 | blood coagulation | 1.75 | 0.0000 |
| GO:0055114 | oxidation-reduction process | 1.53 | 0.0005 |
| GO:0042593 | glucose homeostasis | 2.43 | 0.0005 |
| GO:0030203 | glycosaminoglycan metabolic process | 2.32 | 0.0006 |
| GO:0030168 | platelet activation | 1.93 | 0.0007 |
| GO:0050796 | regulation of insulin secretion | 2.43 | 0.0024 |
| GO:0006024 | glycosaminoglycan biosynthetic process | 3.16 | 0.0026 |
| GO:0005975 | carbohydrate metabolic process | 1.63 | 0.0029 |
| GO:0048010 | vascular endothelial growth factor receptor signaling pathway | 2.24 | 0.0035 |
| GO:0008286 | insulin receptor signaling pathway | 2.01 | 0.0036 |
| GO:0043401 | steroid hormone mediated signaling pathway | 2.48 | 0.0217 |
| GO:0006006 | glucose metabolic process | 1.93 | 0.0259 |
| GO:0000226 | microtubule cytoskeleton organization | 2.21 | 0.0372 |
| GO:0030097 | hemopoiesis | 2.35 | 0.0380 |
| GO:0001568 | blood vessel development | 2.78 | 0.0380 |
| GO:0007507 | heart development | 1.77 | 0.0416 |

Table S2. KEGG enrichment pathways

| Term | Term Name | Foldchange | *P* |
| --- | --- | --- | --- |
| hsa05412 | Arrhythmogenic right ventricular cardiomyopathy ARVC | 2.53 | 0.0005 |
| hsa05414 | Dilated cardiomyopathy | 2.35 | 0.0004 |
| hsa05410 | Hypertrophic cardiomyopathy HCM | 2.11 | 0.0052 |
| hsa04930 | Type II diabetes mellitus | 2.39 | 0.0109 |
| hsa04261 | Adrenergic signaling in cardiomyocytes | 2.47 | 0.0000 |
| hsa04260 | Cardiac muscle contraction | 2.04 | 0.0149 |
| hsa04270 | Vascular smooth muscle contraction | 2.35 | 0.0000 |
| hsa04920 | Adipocytokine signaling pathway | 1.90 | 0.0452 |
| hsa04915 | Estrogen signaling pathway | 2.42 | 0.0001 |
| hsa04922 | Glucagon signaling pathway | 2.05 | 0.0038 |
| hsa04911 | Insulin secretion | 2.67 | 0.0000 |
| hsa04910 | Insulin signaling pathway | 1.86 | 0.0047 |
| hsa04923 | Regulation of lipolysis in adipocytes | 2.12 | 0.0279 |
| hsa00061 | Fatty acid biosynthesis | 3.25 | 0.0464 |
| hsa00561 | Glycolipid metabolism | 2.01 | 0.0462 |
| hsa00564 | Glycerophospholipid metabolism | 2.24 | 0.0014 |
| hsa04152 | AMPK signaling pathway | 2.16 | 0.0004 |
| hsa04010 | MAPK signaling pathway | 2.20 | 0.0000 |
| hsa04151 | PI3K Akt signaling pathway | 1.91 | 0.0000 |

Table S3. Genes selected from previous literatures

| **Authors** | **Genes** |
| --- | --- |
| Qin, 2019(1) | *ABCG1* |
|  | *APOE* |
| Aslibekyan, 2018(2) | *NLRC5* |
|  | *ABO* |
| Iwata, 2016(3) | *PARP14* |
| Sayols-Baixeras, 2016(4) | *CPT1A* |
| Hop, 2020(5) | *NFKBIE* |
|  | *IKZF1* |
|  | *BRD3* |
| Tang, 2014(6) | *BCL11A* |
| Huang, 2017(7) | *PTPN1* |
| Asllanaj, 2020(8) | *KDM6A* |
|  | *GALNT2* |
|  | *INS, INS-IGF2* |
|  | *PON1* |
|  | *CBS* |
|  | *AMT* |
|  | *CCDC8* |
| Pfeiffer, 2015(9) | *TNIP1* |
| Dekkers, 2016(10) | *CPT1A* |
| Peng, 2014(11) | *GALNT2* |
| Boström, 2016(12) | *SKOR2* |
|  | *EHMT2* |


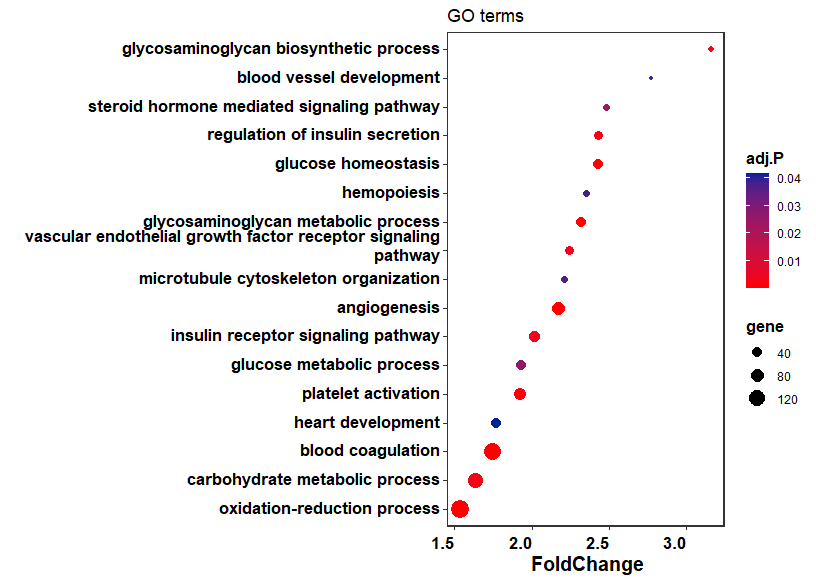


Figure S1. GO enrichment pathways


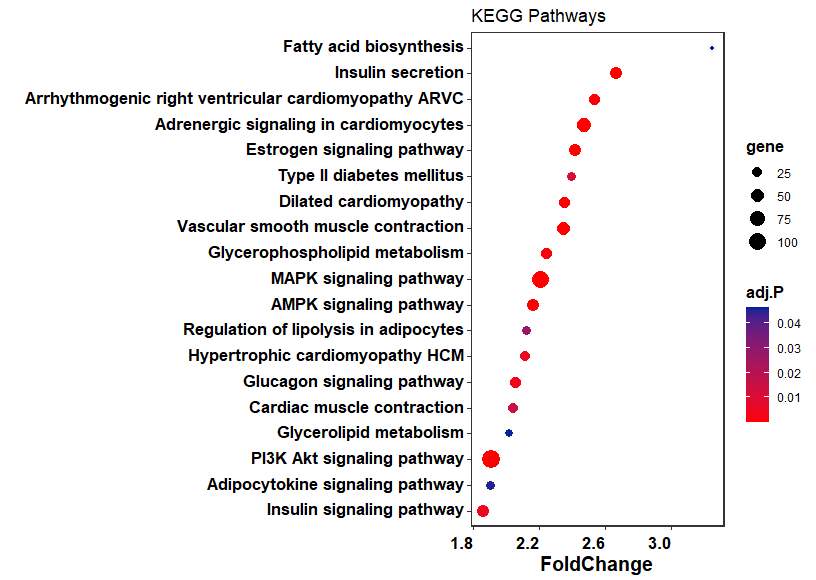


Figure S2. KEGG enrichment pathways

Type 2 diabetes(T2D) patients entered the cohort at baseline

(n= 2756)

T2D patients without CV disease at baseline (n= 1438)

(n=)

**Validation cohort**:

100 CV patients and 100 non-CV patients were randomly selected

**Discovery cohort**:

10 CV patients and 10 non-CV patients matched for sex, age, smoking status, and BMI

CV diseases occurred (n= 210) No CV diseases (n= 1157)

Lost to follow-up (n=71)

With cardiovascular (CV) disease at baseline (n= 1378)

After 6.5 years of follow-up

Figure S3. Flowchart of participants selection

**Reference**

1. Qin X, Li J, Wu T, et al. Overall and sex-specific associations between methylation of the ABCG1 and APOE genes and ischemic stroke or other atherosclerosis-related traits in a sibling study of Chinese population. Clin Epigenetics. 2019;11(1):189.

2. Aslibekyan S, Agha G, Colicino E, et al. Association of Methylation Signals With Incident Coronary Heart Disease in an Epigenome-Wide Assessment of Circulating Tumor Necrosis Factor α. JAMA Cardiol. 2018;3(6):463-72.

3. Iwata H, Goettsch C, Sharma A, et al. PARP9 and PARP14 cross-regulate macrophage activation via STAT1 ADP-ribosylation. Nat Commun. 2016;7:12849.

4. Sayols-Baixeras S, Subirana I, Lluis-Ganella C, et al. Identification and validation of seven new loci showing differential DNA methylation related to serum lipid profile: an epigenome-wide approach. The REGICOR study. Hum Mol Genet. 2016;25(20):4556-65.

5. Hop PJ, Luijk R, Daxinger L, et al. Genome-wide identification of genes regulating DNA methylation using genetic anchors for causal inference. Genome Biol. 2020;21(1):220.

6. Tang L, Wang L, Ye H, et al. BCL11A gene DNA methylation contributes to the risk of type 2 diabetes in males. Exp Ther Med. 2014;8(2):459-63.

7. Huang Q, Han L, Liu Y, et al. Elevation of PTPN1 promoter methylation is a significant risk factor of type 2 diabetes in the Chinese population. Exp Ther Med. 2017;14(4):2976-82.

8. Asllanaj E, Zhang X, Ochoa Rosales C, et al. Sexually dimorphic DNA-methylation in cardiometabolic health: A systematic review. Maturitas. 2020;135:6-26.

9. Pfeiffer L, Wahl S, Pilling LC, et al. DNA methylation of lipid-related genes affects blood lipid levels. Circ Cardiovasc Genet. 2015;8(2):334-42.

10. Dekkers KF, van Iterson M, Slieker RC, et al. Blood lipids influence DNA methylation in circulating cells. Genome Biol. 2016;17(1):138.

11. Peng P, Wang L, Yang X, et al. A preliminary study of the relationship between promoter methylation of the ABCG1, GALNT2 and HMGCR genes and coronary heart disease. PLoS One. 2014;9(8):e102265.

12. Boström AE, Mwinyi J, Voisin S, et al. Longitudinal genome-wide methylation study of Roux-en-Y gastric bypass patients reveals novel CpG sites associated with essential hypertension. BMC Med Genomics. 2016;9:20.
